# Evaluation of Blood–Brain Barrier Disruption Using Low- and High-Molecular-Weight Complexes in a Single Brain Sample in a Rat Traumatic Brain Injury Model: Comparison to an Established Magnetic Resonance Imaging Technique

**DOI:** 10.3390/ijms252011241

**Published:** 2024-10-19

**Authors:** Vladislav Zvenigorodsky, Benjamin F. Gruenbaum, Ilan Shelef, Anat Horev, Abed N. Azab, Anna Oleshko, Mammduch Abu-Rabia, Shahar Negev, Alexander Zlotnik, Israel Melamed, Matthew Boyko

**Affiliations:** 1 Department of Radiology, Soroka University Medical Center, Faculty of Health Sciences, Ben-Gurion University of the Negev, Beer-Sheva 84101, Israel; vladzv@clalit.org.il (V.Z.); shelef@bgu.ac.il (I.S.); 2 Department of Anesthesiology and Perioperative Medicine, Mayo Clinic, Jacksonville, FL 32224, USA; gruenbaum.benjamin@mayo.edu; 3 Department of Neurology, Soroka University Medical Center, Ben-Gurion University of the Negev, Beer-Sheva 84101, Israel; anatho@bgu.ac.il; 4 Department of Nursing, Recanati School for Community Health Professions, Faculty of Health Sciences, Ben-Gurion University of the Negev, Beer-Sheva 84101, Israel; azab@bgu.ac.il; 5 Department of Biology and Methods of Teaching Biology, A. S. Makarenko Sumy State Pedagogical University, 40002 Sumy, Ukraine; oleshko@post.bgu.ac.il; 6 Department of Anesthesiology and Critical Care, Soroka University Medical Center, Ben-Gurion University of the Negev, Beer-Sheva 84101, Israel; mamdohab@clalit.org.il (M.A.-R.); negevs@post.bgu.ac.il (S.N.); zlotnika@bgu.ac.il (A.Z.); 7 Department of Neurosurgery, Soroka University Medical Center, Faculty of Health Sciences, Ben-Gurion University of the Negev, Beer-Sheva 84101, Israel; melamedi@bgu.ac.il

**Keywords:** animal model, blood–brain barrier, high-molecular-weight complex, low-molecular-weight complex, magnetic resonance imaging, traumatic brain injury

## Abstract

Traumatic brain injury (TBI), a major cause of death and disability among young people, leads to significant public health and economic challenges. Despite its frequency, treatment options remain largely unsuitable. However, examination of the blood–brain barrier (BBB) can assist with understanding the mechanisms and dynamics of brain dysfunction, which affects TBI sufferers secondarily to the injury. Here, we present a rat model of TBI focused on two standard BBB assessment markers, high- and low-molecular-weight complexes, in order to understand BBB disruption. In addition, we tested a new technique to evaluate BBB disruption on a single brain set, comparing the new technique with neuroimaging. A total of 100 Sprague–Dawley rats were separated into the following five groups: naive rats (*n* = 20 rats), control rats with administration (*n* = 20 rats), and TBI rats (*n* = 60 rats). Rats were assessed at different time points after the injury to measure BBB disruption using low- and high-molecular-weight complexes. Neurological severity score was evaluated at baseline and at 24 h following TBI. During the neurological exam after TBI, the rats were scanned with magnetic resonance imaging and euthanized for assessment of the BBB permeability. We found that the two markers displayed different examples of BBB disruption in the same set of brain tissues over the period of a week. Our innovative protocol for assessing BBB permeability using high- and low-molecular-weight complexes markers in a single brain set showed appropriate results. Additionally, we determined the lower limit of sensitivity, therefore demonstrating the accuracy of this method.

## 1. Introduction

Traumatic brain injury (TBI) is a leading contributor to death and disability in children and young adults [1]. Causing up to a third of all accidental deaths and approximately two-thirds of all hospital trauma-related deaths [2], TBI has a tremendous impact on public health and socioeconomic growth worldwide [3,4]. TBI, even if the trauma is mild, often causes lifelong disability in sufferers, along with long-term impacts on the healthcare system [5,6]. Despite targeted efforts to treat TBI and its related secondary complications, current treatment options are not always effective [7].

Animal models are necessary to better understand the pathophysiology of TBI and to advance the development of new therapies targeted at reducing and repairing neurological damage. Highly precise assessments of parameters of brain tissue destruction and behavioral outcomes are vital to the success of these models. Of these, histological evaluation, neuroimaging, and neurological assessments are the most common techniques used in evaluating TBI in rodent models [8,9]. BBB assessment is one of the three major histological outcomes of TBI in rats, along with cerebral edema and lesion volume [8]. It is well known that histological assessment remains the gold standard for assessing the outcome of TBI in rats to determine its severity. Previously, we have shown that BBB permeability is the most sensitive among other histological tests [8] and that, in the human population, BBB breakdown is considered a major risk factor for high mortality and morbidity [10]. Thus, focusing on the role of the BBB in acute and chronic sequelae after TBI will help to develop new diagnostic methods, as well as prognostic and therapeutic strategies.

The BBB of the vertebrae has a complex structure that is heterogenous and consists of many cellular components that work together to isolate the CNS from systemic circulation [11]. Among its functions are protecting the fragile homeostasis of the CNS from blood-borne neurotoxic and inflammatory risks, assisting with clearing metabolic by-products from the brain, modulating a specifically organized extracellular matrix, and overseeing CNS and periphery communication with immune cells and soluble factors [12]. As a result, BBB dysfunction is a factor in acute brain disorders such as ischemic and hemorrhagic stroke, traumatic brain injury, cerebral malaria, and other central and systemic infections [13,14]. These conditions involve edema and neurotoxicity caused by an influx of blood products into the brain parenchyma, attributable to BBB damage. In addition, BBB dysfunction has been recently noted as a factor in chronic neurogenerative disorders including Alzheimer’s disease, cerebral small vessel disease, and multiple sclerosis; it is also believed to contribute directly to cognitive impairment [15,16,17]. Oxidative stress also plays a crucial role in BBB disruption following TBI, as it impairs endothelial tight junctions and promotes edema [18]. Additionally, oxidative stress-induced excitotoxicity further exacerbates neuronal damage and BBB compromise [19]. Recent studies also suggest that oxidative stress can influence cellular signaling pathways, which may pave the way for novel biomarker discovery in the context of TBI and BBB dysfunction [20,21].

In our recent study, we proposed a revolutionary hypothesis about chronic disruption of the BBB, which causes chronic neurotoxicity and ultimately leads to the development of a number of neurodegenerative diseases [22]. We subsequently confirmed this hypothesis in preclinical studies [23,24,25,26] and human studies using large patient populations [27,28]. We anticipate that increased knowledge about the differences between a healthy and a damaged BBB will assist with our ability to identify, catalogue, and treat the many debilitating conditions affected by BBB dysfunction [29].

Recent studies indicate that regaining BBB health can take 1–3 months [30] or even up to 10 months [31] in rats and many years in humans [10,32,33]. Accurate and reliable methods for assessing BBB permeability are critical for studying various brain injuries and their related conditions and for ultimately developing new treatment strategies. These methods provide us with information about the mechanisms and dynamics of BBB disruption and help us evaluate the effectiveness of drug testing. The complexity of the BBB and the array of diseases in which several components are affected necessitates a multimodal approach to imaging these changes.

In a healthy brain, the BBB has a stringent system of diffusion. The only molecules able to pass the BBB must satisfy Lipinski’s Rule of Five as follows: They must be smaller than 500 Da, have less than five hydrogen bond donors and ten hydrogen bond acceptors, and have an octanol–water partition coefficient of less than or equal to five [34,35]. Specific processes maintained by receptors and transporters allow some large or polar molecules to enter a healthy BBB as well. These properties are maintained by the interactions between several types of cells and molecular mediators in a healthy system and are responsible for disruptions in inflammatory and pathological conditions.

When this system is disrupted in one of two ways, the BBB becomes dysfunctional [10]. One disruption involves increased paracellular transport occurring after a loss of tight junction (TJ) proteins, which introduces molecules into the BBB that normally cannot pass [36,37], including immune cells such as neutrophils that can worsen inflammatory effects. The second disruption happens when transcytosis is increased across the endothelial cells (ECs), during which larger molecules and serum proteins such as albumin, normally without access to the brain, can enter [36,37].

A number of methods for assessing BBB permeability have been described in the literature [11,12,38,39,40]. Low-molecular-weight complexes such as gadolinium (molecular weight of 552 Da) [41] and sodium fluorescein (FNa) (molecular weight of 376 Da) [42] are typically imaging markers for solute and ion permeability, while high-molecular-weight complexes such as Evans blue (EB) dye, which binds to albumin (molecular weight, ≈68 kDa), are imaging markers for protein permeability [43,44]. Evans blue and sodium fluorescein are considered the most popular and simple methods for measuring BBB permeability in animal models [45]. In addition, sodium fluorescein is considered the most accurate research method due to its ability to be measured in low concentrations [12].

We previously developed an alternative protocol to histologically examine BBB breakdown, brain edema, and lesion volume in rats following TBI in the same set of brain samples and compared it with neuroimaging [8]. Here, we studied the dynamics of BBB disruption using the two most popular BBB assessment markers, high- and low-molecular-weight complexes, using a rat model of TBI. The second goal of our study was to establish and test a new technique for evaluating BBB disruption on one brain set by using these markers to assess the permeability of BBB for high- and low-molecular-weight complexes and then comparing the results with another neuroimaging technique.

We believe that this study will contribute to a deeper understanding of the mechanisms of BBB disruption in TBI by providing a complete picture of BBB permeability at a critical time point for both low- and high-molecular-weight complexes, with the ultimate goal of the safe and effective treatment of brain injury.

## 2. Results

### 2.1. Neurological Severity Score (NSS)

There was no evidence of neurological deficit in the naive and control group. The NSS at 24 h was significantly greater in TBI rats compared to the control group (4(2–6) vs. 0(0–0), U = 20, *p* < 0.01, r = 0.85). The data are measured as a count and expressed as median and 25–75 percentile range (Mann–Whitney U test). No significant difference was observed between males and females.

### 2.2. Analysis of BBB Disruption by MRI Method

**Figure 1:**
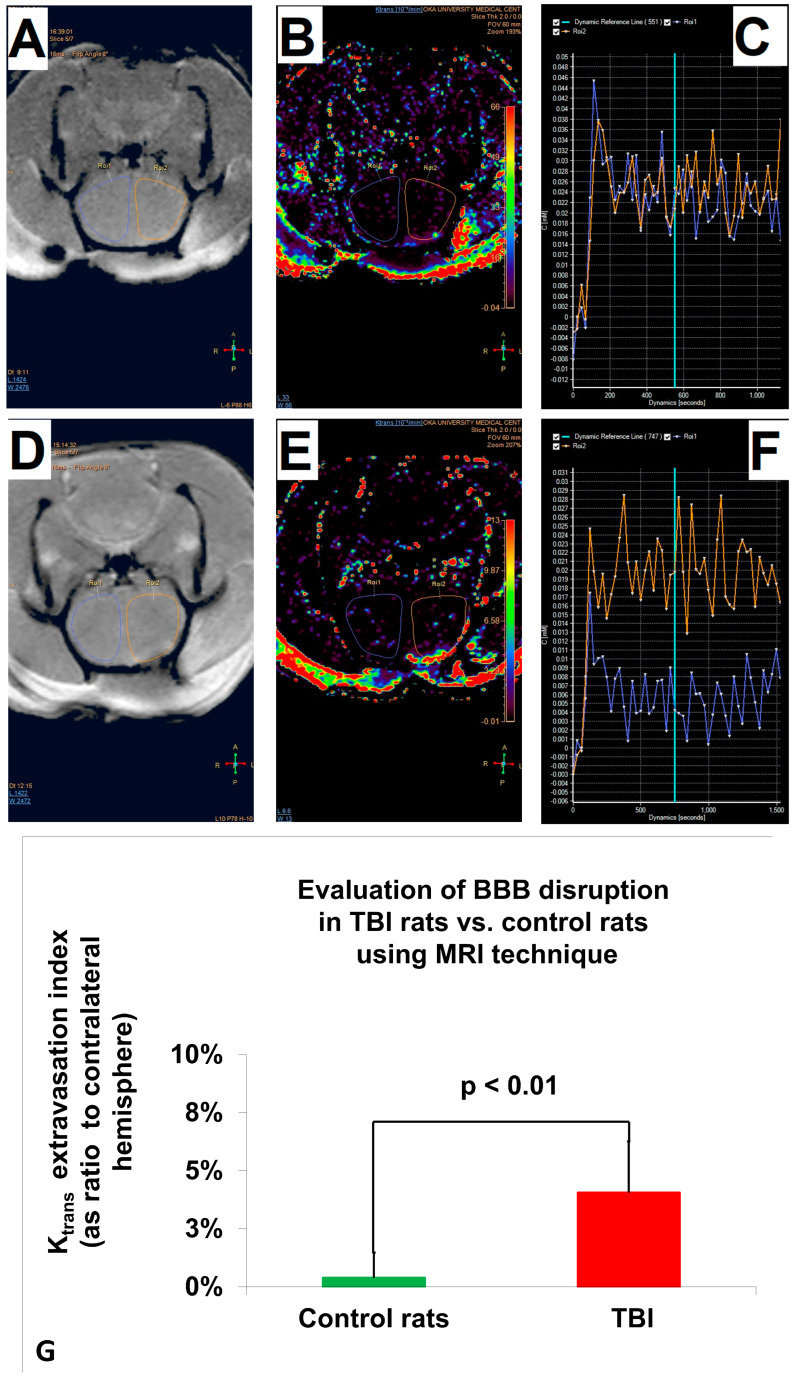
Illustrated assessment of BBB disruption in TBI rats vs. control rats using MRI. (**A**) Sample MRI images of the brain in a control rat, with regions of interest (ROI) outlined. (**B**) Example of color-coded maps representing BBB permeability, visualizing the calculated Ktrans in control rats. There is a noticeable increase in permeability in the TBI rats compared to the control, as indicated by the color intensity. (**C**) Example of dynamics of contrast agent flow over time in control rats, with lines corresponding to the rate of contrast agent extravasation. (**D**) Sample MRI images of the brain in a TBI rat, with ROI outlined. (**E**) Example of color-coded maps representing BBB permeability, visualizing the calculated Ktrans in TBI rats. (**F**) Example of dynamics of contrast agent flow over time in TBI rats, with lines corresponding to the rate of contrast agent extravasation. Note that the TBI rats show higher and more variable values over time compared to controls, indicating greater BBB disruption. (**G**) Ktrans extravasation index (expressed as a ratio of the affected to contralateral hemisphere) for both control (green) and TBI (red) rats. The TBI group showed a significantly higher extravasation index compared to controls, demonstrating a marked increase in BBB permeability.

The data were analyzed with a two-way ANOVA to determine the effect of the brain trauma (TBI or sham) and sex (male or female) on BBB permeability (K_trans_). The two-way ANOVA showed a significant effect of trauma (F_1,36_ = 43.3, *p* < 0.01, pη^2^ = 0.546). No effects of sex or interaction between sex and trauma on K_trans_ were found. The analysis using Student’s *t*-test showed significantly higher K_trans_ in the 20 TBI rats than the 20 control rats at 24 h after injury (4.06% ± 2.2%, $\bar{x}$ ± SD, vs. 0.39% ± 1.09%, $\bar{x}$ ± SD; t(27.7) = 6.7; *p* < 0.01; d = 2.11). Levene’s test indicated unequal variances (F = 17.75, *p* < 0.05), so the degrees of freedom were adjusted from 38 to 27.7. The data are measured as ratio to contralateral hemisphere and expressed as a percentage, presented as mean ± SD (see Figure 1).

### 2.3. Analysis of BBB Disruption Using a Histological Method

#### 2.3.1. Analysis of BBB Permeability Using a Low-Molecular-Weight Complex Marker

**Figure 2:**
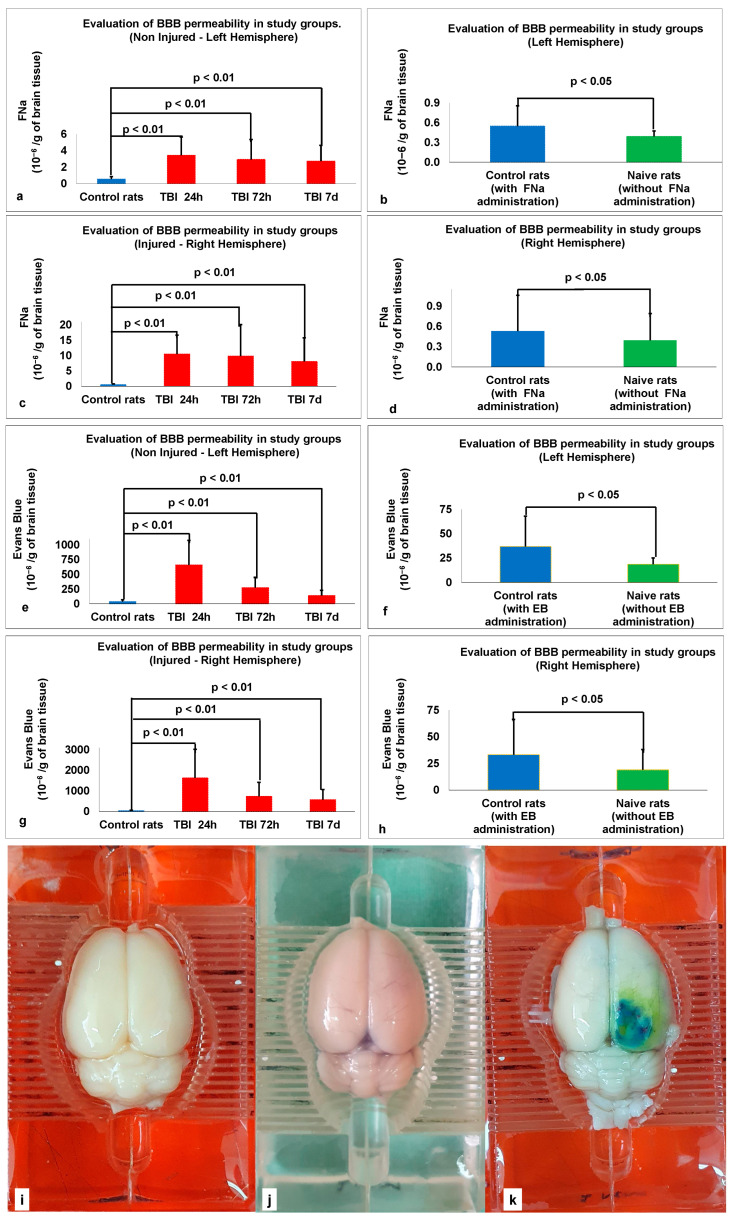
Illustrated assessment of BBB disruption in the study groups using a histological method. (**a**) FNa extravasation between control rats and rats after TBI at 24 h, 72 h, and 7 d in the non-injured left hemisphere. (**b**) FNa extravasation between control rats and naïve rats in the left hemisphere. (**c**) FNa extravasation between control rats and rats after TBI at 24 h, 72 h, and 7 d in the injured right hemisphere. (**d**) FNa extravasation between control rats and naïve rats in the right hemisphere. (**e**) Evans Blue extravasation between control rats and rats after TBI at 24 h, 72 h, and 7 d in the non-injured left hemisphere. (**f**) Evans Blue extravasation between control rats and naïve rats in the left hemisphere. (**g**) Evans Blue extravasation between control rats and rats after TBI at 24 h, 72 h, and 7 d in the injured right hemisphere. (**h**) Evans Blue extravasation between control rats and naïve rats in the right hemisphere. (**i**) Representative histological image of a naive rat. (**j**) Representative histological image of a control rat. (**k**) Representative histological image of a TBI rat.

In the non-injured (left) hemisphere, the data were analyzed with a two-way ANOVA to determine the effect of the brain trauma (TBI 24 h, TBI 72 h, TBI 7 d, control and naive rats) and sex (male or female) on BBB permeability (FNa). The two-way ANOVA showed a significant effect of trauma (F_4,90_ = 13.4, *p* < 0.01, pη^2^ = 0.374). No effects of sex or interaction between sex and trauma on FNa were found. The analysis using Student’s t-test showed a significant difference in the FNa extravasation between the (i) 20 TBI rats 24 h after injury (3.43 ± 2.21, $\bar{x}$ ± SD; t(19.75) = 5.8; *p* < 0.01; d = 1.83; Levene’s test indicated unequal variances, F = 23.33, *p* < 0.05, so degrees of freedom were adjusted from 38 to 19.75); (ii) 20 TBI rats 72 h after injury (2.93 ± 2.4, $\bar{x}$ ± SD; t(19.63) = 4.4; *p* < 0.01; d = 1.39; Levene’s test indicated unequal variances, F = 28.9, *p* < 0.05, so degrees of freedom were adjusted from 38 to 19.63); (iii) 20 TBI rats 7 d after injury (2.72 ± 1.91, $\bar{x}$ ± SD; t(20) = 5.03; *p* < 0.01; d = 1.59; Levene’s test indicated unequal variances, F = 45.9, *p* < 0.05, so degrees of freedom were adjusted from 38 to 20); (iv) 20 naive rats (0.39 ± 0.08, $\bar{x}$ ± SD; t(21.7) = 2.18; *p* < 0.05; d = 0.7; Levene’s test indicated unequal variances, F = 19.9, *p* < 0.05, so degrees of freedom were adjusted from 38 to 21.7) and the 20 control rats (0.55 ± 0.31, $\bar{x}$ ± SD). The data are expressed as 10^−6^/g of brain tissue and presented as mean ± SD (see Figure 2a,b).

In the injured (right) hemisphere, the data were analyzed with a two-way ANOVA to determine the effect of the brain trauma (TBI 24 h, TBI 72 h, TBI 7 d, control, and naive rats) and sex (male or female) on BBB permeability (FNa). A two-way ANOVA showed a significant effect of trauma (F_4,90_ = 11.8, *p* < 0.01, pη^2^ = 0.344). No effects of sex or interaction between sex and trauma on FNa were found. The analysis using Student’s *t*-test showed a significant difference in the FNa extravasation between the (i) 20 TBI rats 24 h after injury (10.5 ± 6.19, $\bar{x}$ ± SD; t(19.1) = 7.2; *p* < 0.01; d = 2.28; Levene’s test indicated unequal variances, F = 53.7, *p* < 0.05, so degrees of freedom were adjusted from 38 to 19.1); (ii) 20 TBI rats 72 h after injury (9.85 ± 10.24, $\bar{x}$ ± SD; t(19.02) = 4.07; *p* < 0.01; d = 1.29; Levene’s test indicated unequal variances, F = 18.27, *p* < 0.05, so degrees of freedom were adjusted from 38 to 19.02); (iii) 20 TBI rats 7 d after injury (8.07 ± 7.67, $\bar{x}$ ± SD; t(19.04) = 4.39; *p* < 0.01; d = 1.39; Levene’s test indicated unequal variances, F = 23.6, *p* < 0.05, so degrees of freedom were adjusted from 38 to 19.04); (iv) 20 naive rats (0.4 ± 0.1, $\bar{x}$ ± SD; t(24.9) = 2.23; *p* < 0.05; d = 0.68; Levene’s test indicated unequal variances, F = 16.8, *p* < 0.05, so degrees of freedom were adjusted from 38 to 24.9) and the 20 control rats (0.53 ± 0.25, $\bar{x}$ ± SD). The data are expressed as 10^−6^/g of brain tissue and presented as mean ± SD (see Figure 2c,d).

#### 2.3.2. Analysis of BBB Permeability Using a High-Molecular-Weight Complex Marker

In the non-injured (left) hemisphere, the data were analyzed with a two-way ANOVA to determine the effect of the brain trauma (TBI 24 h, TBI 72 h, TBI 7 d, control, and naive rats) and sex (male or female) on BBB permeability (EB). The two-way ANOVA showed a significant effect of trauma (F4,90 = 30.8, *p* < 0.01, pη2 = 0.578). No effects of sex or interaction between sex and trauma on EB were found. The analysis using Student’s t-test showed a significant difference in the EB extravasation between the (i) 20 TBI rats 24 h after injury (659 ± 419, $\bar{x}$ ± SD; t(19.21) = 6.6; *p* < 0.01; d = 2.1; Levene’s test indicated unequal variances, F = 42.2, *p* < 0.05, so degrees of freedom were adjusted from 38 to 19.21); (ii) 20 TBI rats 72 h after injury (275 ± 174, $\bar{x}$ ± SD; t(20.23) = 6.05; *p* < 0.01; d = 1.91; Levene’s test indicated unequal variances, F = 37.7, *p* < 0.05, so degrees of freedom were adjusted from 38 to 20.23); (iii) 20 TBI rats 7 d after injury (140 ± 88, $\bar{x}$ ± SD; t(23.75) = 5; *p* < 0.01; d = 1.57; Levene’s test indicated unequal variances, F = 20.4, *p* < 0.05, so degrees of freedom were adjusted from 38 to 23.75); (iv) 20 naive rats (18.5 ± 6.5, $\bar{x}$ ± SD; t(20.7) = 2.55; *p* < 0.05; d = 0.81; Levene’s test indicated unequal variances, F = 6.67, *p* < 0.05, so degrees of freedom were adjusted from 38 to 20.7) and the 20 control rats (36.7 ± 31.2, $\bar{x}$ ± SD). The data are expressed as 10^−6^/g of brain tissue and presented as mean ± SD (see Figure 2e,f).

In the injured (right) hemisphere, the data were analyzed with a two-way ANOVA to determine the effect of the brain trauma (TBI 24 h, TBI 72 h, TBI 7 d, control, and naive rats) and sex (male or female) on BBB permeability (EB). The two-way ANOVA showed a significant effect of trauma (F4,90 = 15.9, *p* < 0.01, pη2 = 0.415). No effects of sex or interaction between sex and trauma on EB were found. The analysis using Student’s t-test showed a significant difference in the EB extravasation between the (i) 20 TBI rats 24 h after injury (1633 ± 1388, $\bar{x}$ ± SD; t(19) = 5.15; *p* < 0.01; d = 1.63; Levene’s test indicated unequal variances, F = 71.5, *p* < 0.05, so degrees of freedom were adjusted from 38 to 19); (ii) 20 TBI rats 72 h after injury (739 ± 671, $\bar{x}$ ± SD; t(19.04) = 4.7; *p* < 0.01; d = 1.49; Levene’s test indicated unequal variances, F = 26.4, *p* < 0.05, so degrees of freedom were adjusted from 38 to 19.04); (iii) 20 TBI rats 7 d after injury (575 ± 487, $\bar{x}$ ± SD; t(19.07) = 4.97; *p* < 0.01; d = 1.57; Levene’s test indicated unequal variances, F = 26.7, *p* < 0.05, so degrees of freedom were adjusted from 38 to 19.07), (iv) 20 naive rats (19 ± 8.6, $\bar{x}$ ± SD; t(24.98) = 2.75; *p* < 0.05; d = 0.87; Levene’s test indicated unequal variances, F = 17.4, *p* < 0.05, so degrees of freedom were adjusted from 38 to 24.98) and the 20 control rats (33.2 ± 21.4, $\bar{x}$ ± SD). The data are expressed as 10^−6^/g of brain tissue and presented as mean ± SD (see Figure 2g,h). Representative histological images are shown in Figure 2i–k.

### 2.4. Analysis of Specificity and Determination of the Lower Limit of Sensitivity

**Figure 3:**
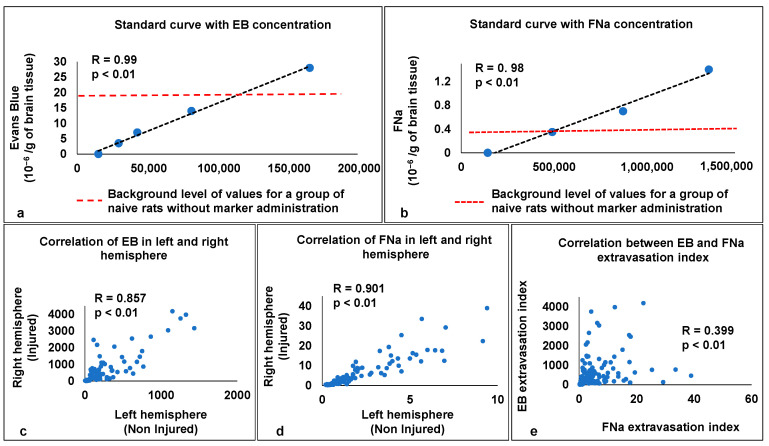
Standard curve for EB and FNa and correlation between permeability markers measurements in the study groups. (**a**) Standard curve of EB with concentrations in well from 0 × 10^−6^/g of brain tissue to 28 × 10^−6^/g brain tissue, equivalent to the content in brain tissue. (**b**) Standard curve of FNa with concentrations in well from 0 × 10^−6^/g of brain tissue to 3.5 × 10^−6^/g brain tissue, equivalent to the content in brain tissue. The red line shows the autofluorescence level of values in brain tissue in the naive group without marker administration. (**c**) Correlation between the results of the EB extravasation index when comparing the left and right hemispheres in the study groups. (**d**) Correlation between the results of the FNa extravasation index when comparing the left and right hemispheres in the study groups. (**e**) Correlation between the results of the EB extravasation index and the FNA extravasation index in the study groups.

One of the objectives of this study was to determine the lower limit of marker concentration in the brain tissue using this new method. For this purpose, a spectrophotometer was used to determine the background level of the wavelength values for each marker in the brain tissue (“Control rats (with the introduction of EB or FNa)” and “Naive rats (without the introduction of EB or FNa)”), which is a natural limiter of the measurement of marker concentrations at concentrations below this value. We also determined the lower limits of the concentration curve for both markers, at which accurate determination of marker concentrations in brain tissue is possible (see Figure 3).

The standard curve of EB with concentrations from 0 × 10^−6^/g of brain tissue to 28 × 10^−6^/g brain tissue and FNa with concentrations from 0 × 10^−6^/g of brain tissue to 3.5 × 10^−6^/g brain tissue showed a high correlation of [r(4) = 0.98, *p* < 0.01] and [r(5) = 0.99, *p* < 0.01], respectively. The values for the naive group without marker administration for the EB marker ($\bar{x}$ = 19) and the FNa marker ($\bar{x}$ = 0.4) were within the standard curve (see Figure 3a,b, red line).

### 2.5. Correlational Analysis

MRI techniques for assessing the post-TBI BBB permeability were compared to histological techniques. No correlation was found between the EB extravasation index [r(40) = 0.02, *p* = non-significant] or the FNa extravasation index [r(40) = 0.15, *p* = non-significant] and the K_trans_-MRI at 24 h post-injury. High correlation was found in the EB extravasation index [r_(100)_ = 0.857, *p* < 0.01] and FNa extravasation index [r_(100)_ = 0.901, *p* < 0.01] results when comparing the left and right hemispheres in the experimental groups (see Figure 3c,d). Low correlation was found between EB extravasation index and FNa extravasation index [r(200) = 0.399, *p* < 0.01] in study groups (Figure 3e).

## 3. Discussion

The objectives of this study were to study the dynamics of BBB disruption using the two most popular BBB assessment markers, high- and low-molecular-weight complexes, in a rat model of TBI. The second goal of our study was to establish and test a new technique for evaluating BBB disruption on one brain set using those two markers to assess the permeability of BBB and to compare the results with neuroimaging techniques.

The main finding of this study was that the above-mentioned markers demonstrated different dynamics of BBB disruption over a week in the same set of brain tissue using a TBI model. We developed a protocol for assessing BBB permeability using high- and low-molecular-weight complexes as markers in a single brain set, and we further determined the lower limit of sensitivity and demonstrated the accuracy of this method.

As expected, the results showed BBB disruption assessed by the MRI protocol after 24 h and the dynamics of BBB disruption measured by two histological markers, EB and FNa dyes, over the course of one week. BBB disruption detected by the EB marker showed a maximum peak at 24 h and then a decrease in concentration over the course of a week in the affected hemisphere. A similar pattern was observed in the contralateral hemisphere, but the marker concentration was significantly lower. We have already recorded this phenomenon in our previous publication [8].

We suggest that there are two mechanisms causing this phenomenon. The first is the impact of the injury itself on the contralateral hemisphere, caused by both the impact itself, which is transmitted to the entire brain tissue, and the force within the closed walls of the skull, as well as by the secondary contact of the healthy hemisphere with the opposite wall of the skull. It is also well known that BBB disruption can cause and influence cerebral edema in TBI [8], so it can be hypothesized that TBI-induced cerebral edema also contributes to increased BBB permeability in the control lateral hemisphere [12]. However, in our previous studies using laser brain injury models [46], where there was no traumatic impact on brain tissue and the effect of damage to the contralateral hemisphere was minimal, we also noted this phenomenon of increased blood–brain barrier permeability in the contralateral hemisphere. Since the BBB disruption we recorded in the contralateral hemisphere was of high intensity, which is difficult to attribute only by the potential influence of cerebral edema, we assume that the contralateral hemisphere is involved in the mechanisms of purification and drainage of toxic substances entering the cerebrospinal and extracellular fluid from the site of damage (the ipsilateral hemisphere). This may explain the presence of markers not only in the affected hemisphere and cerebrospinal fluid, where they penetrated from the damaged area, but also in healthy areas of the contralateral hemisphere, through which toxins are excreted. Thus, the assessment of permeability in the affected hemisphere shows the extent of the lesion, and the assessment of permeability in the ipsilateral hemisphere also shows the efficiency of the efflux systems. The effect of increased permeability in the healthy hemisphere was also recorded using the FNa marker, but its trajectory was completely different from that of the EB one. The FNa marker, similar to EB, showed an increase 24 h after injury but did not decrease like EB. Instead, it plateaued, remaining at almost the same values for a week after injury.

EB and FNa were administered to rats at a concentration of 0.02 mol/l in 4 mL/kg saline 0.9%. However, the recorded group means at the same time points were different. The recorded EB concentrations were significantly higher than FNa concentrations. The explanation for this phenomenon lies in the different pharmacokinetics and pharmacodynamics of these markers [47]. It is known that EB binds to albumin and other tissues of the body, the lifespan of which is measured over a long period of time [45]. Thus, EB can be detected in the blood in high concentrations many days after ingestion [48] unlike FNa, which is excreted from the body by the kidneys much more quickly [47].

Another goal of our study was to produce and test a new method for assessing BBB disruption on a single set of brains using low- and high-molecular-weight complex markers. To do this, we needed to determine the lower limits of sensitivity of the concentration curve and the levels of autofluorescence values of brain samples that could be considered false positive results. The autofluorescence values in the group of naive rats without the administration of markers determined the lower limit of the fluorospectrometry capabilities of this protocol. The concentration curve effectively assessed the marker concentrations, starting from the autofluorescence boundary levels (in the naive group without marker introduction) and above. Control rats with an intact BBB that were injected with EB and FNa showed significantly higher levels of these markers in brain tissue compared to naive rats without marker administration. The permeability of an intact BBB by EB and FNa markers was previously investigated and documented [48]. Thus, in studies measuring BBB permeability using the fluorescence method, it is useful to show not only the control groups that were injected with a marker for permeability testing (EB or FNa) but also the level of autofluorescence in naive rats that were not injected with the marker.

Correlational analysis showed a strong correlation between the contralateral and ipsilateral hemispheres for both EB and FNa markers, but an analysis between the markers showed a weak correlation between them. This phenomenon did not surprise us since, in the introduction, we showed in detail that the BBB is a complex multi-level system that is designed to protect the brain from toxins, both by filtering and controlling the passage of useful components and by removing toxins in the event of penetration into the brain compartment. And since the markers we study differ in molecular weight and molecular diameter, and the trajectory of their detection in the affected brain also differs, it is not surprising that the correlation we found between them was weak.

We initially anticipated a stronger correlation between the MRI-based assessments of BBB permeability and histological measurements using low-molecular-weight markers. However, our correlation analysis did not yield a significant relationship (*p* > 0.05). This discrepancy likely stems from the inherent differences between the two methods. The MRI-derived Ktrans values reflect the contrast agent flow between both the diseased and healthy hemispheres, while histological methods assess BBB permeability in only one hemisphere, leading to differing results. Furthermore, an MRI is generally less sensitive than histological techniques, which may further contribute to the weak correlation observed between the two approaches.

The rationale for using both EB and FNa in our study was due to their differing molecular weights, allowing us to assess BBB permeability to both large and small molecules, providing a more comprehensive evaluation. However, we recognize that neither marker is used in clinical practice for humans. The clinical application of this technique may involve substituting these markers with those more relevant to human medicine. For example, gadolinium [49], a commonly used MRI contrast agent, could serve as a low-molecular-weight marker for BBB disruption, while the albumin index or albumin quotient [50,51], a well-established BBB disruption marker, could represent a high-molecular-weight marker.

It is important to note that there is variability in the reported values of the molecular markers like EB and FNa in the literature. This variability can be attributed to differences in the experimental protocols, including variations in marker concentration, administration route, circulation time, and tissue preparation methods. For example, EB doses in previous studies range from as low as 0.004% [52] to 8% [53], and administration routes vary from intravenous to intraperitoneal [53,54]. These discrepancies, along with differences in tissue homogenization and spectrometry techniques [55], underscore the importance of standardizing the methods for BBB evaluation in future studies.

The effect of gender on BBB permeability has been described in the literature [56]. Some studies have indicated that these differences are more pronounced in specific brain regions and at older ages [57]. For example, a study of 186 individuals found sex-based differences in permeability but only after the age of 62 and only in certain brain areas [58]. BBB dysfunction is also linked to neurodegenerative diseases, which often exhibit sex-specific prevalence and progression patterns [22,59]. While these findings support the hypothesis of sex differences in BBB permeability, they suggest that multiple factors, such as age and brain region, are necessary to observe these differences. In our study, no significant sex differences in BBB permeability were detected, likely due to the experimental model, the small sample size, and the absence of confounding factors such as age.

## 4. Materials and Methods

The experiments were carried out following the guidelines of the Declarations of Helsinki and Tokyo, as well as the European Community’s Guidelines for the Use of Experimental Animals. Approval for the experiments was granted by the Animal Care Committee at Ben-Gurion University of the Negev, Beer-Sheva, Israel.

### 4.1. Animals

The experiments were conducted in a total of 100 Sprague–Dawley rats (Harlan Laboratories, Jerusalem, Israel) weighing between 280 and 320 g each. Purina Chow and water were available ad libitum. Rats were maintained in 12 h:12 h light–dark conditions, at a constant temperature of 22 °C ± 1 °C. All experiments were conducted in the dark phase, between 08:00 and 16:00. Four rats were sacrificed due to postoperative complications such as seizures, surgery-related distress symptoms, or weight loss greater than 15% of baseline after surgery.

### 4.2. Experimental Design

**Figure 4:**
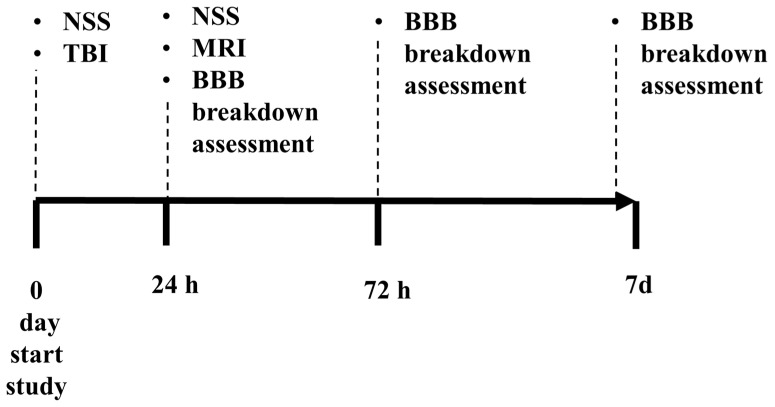
Experimental timeline. BBB—blood–brain barrier; MRI—magnetic resonance imaging; NSS—neurological severity score. TBI—traumatic brain injury.

All rats were randomly separated into 5 groups of 20 rats each (10 males and 10 females) as follows: naive rats without EB and FNa administration (*n* = 20 rats), control rats with EB and FNa administration (*n* = 20 rats), and TBI rats (*n* = 60 rats). The effect of BBB disruption after TBI using low- and high-molecular-weight complex markers was studied in the rats at the following time points after injury: (1) 24 h; (2) 72 h; and (3) 7 days (see Figure 4). Neurological severity was evaluated at baseline before intervention and at 24 h following TBI. Following a neurological assessment at 24 h, all the rats were scanned using a clinical MRI scanner and then euthanized for assessment of BBB permeability at group-appropriate time points (Figure 4).

### 4.3. Neurological Severity Score (NSS)

Two blinded observers noted the NSS scores, as previously described [8,60,61], based on observations of altered motor function and behavior. The combined score ranges from 0 for an intact neurological condition to 15 for the most severe neurological dysfunction and is assigned based on scoring of tasks including the following: gait on a wide surface (3-point scale), gait on a narrow surface (4-point scale), effort to remain on a narrow surface (2-point scale), walking on beam (3-point scale), and balance on beam(3-point scale).

### 4.4. Induction of TBI

The apparatus was previously described by our laboratory [8,23,24,28,62]. Rats received 5% inhaled isoflurane for anesthesia, maintained at 1.5–2.5%, with 50% medical air and 50% oxygen. They were injected with 0.5% bupivacaine into the scalp prior to incision. The scalp was incised and reflected laterally with the left temporal muscle, and the underlying periosteum was dissected to open the skull. A craniotomy (4 mm lateral and 4 mm posterior to bregma) was performed at 5 mm with a trephine (Roboz Surgical Instrument Co., Gaithersburg, MD, USA) affixed to an electrical drill (Stoelting, Wood Dale, IL, USA). A Luer three-way stopcock was adhered with cyanoacrylate adhesive and dental acrylic. The injury was induced by a fluid-percussion device over 21–23 ms through the three-way stopcock, at a pressure pulse of 2.2 atmospheres. A continuous flow of saline allowed for efficient transport of the pressure pulse. After induction, the incision was closed, and the rats were allowed to recover from anesthesia. This experiment involved 2 control groups of rats that did not have BBB disruption, where one group received intravenous EB and FNa and the other group received intravenous saline. A timeline protocol can be seen in Figure 4.

### 4.5. Assessment of BBB Permeability by MRI Technique

An MRI was used for the determination of the volume transfer constant (K_trans_), at 24 h following TBI, which is considered to be an index of BBB permeability, as described previously [8]. Animals were maintained under general anesthesia (1.5% isoflurane in oxygen). A tail vein catheter was introduced and connected to a syringe containing a solution of Gadopentetic acid (Gd-DTPA) (Dotarem, 0.5 mmol/mL Guerbet, Roissy, France). A 3 Tesla (3T) MRI was used (Ingenia, Philips Medical Systems, Best, The Netherlands) using an eight-channel receive-only coil. T1 permeability studies were performed using a segmented 3D T1w-FFE sequence with 50 dynamics for a total scan time of 25:52 min. The scan parameters were TR/TE = 16/4.9 ms turbo factor = 48, SENSE factor 1.5, resolution (freq × phase × slice) = 0.30 × 0.37 × 2.0 mm, tip angle = 80, and two signal averages for a scan time of 31 sec/dynamic. Three calibration scans with identical resolution preceded the dynamic sequence with tip angles of 50, 100, and 150. The contrast agent was injected after the fifth dynamic scan. The Intellispace Portal workstation (V5.0.0.20030, Philips Medical Systems, Best, The Netherlands) was used for the post-processing of the permeability studies.

### 4.6. Assessment of BBB Permeability by Histological Technique

To measure the BBB permeability, the rats were intravenously injected with EB and FNa in the amount of 0.02 mol/l 4 mL/kg. After 1 h, the rats were euthanized, and the brain was removed to assess the BBB permeability. To remove the intravascularly localized dye, the rats’ chests were opened, and the animals were perfused with cooled saline through the left ventricle at a pressure of 110 mm Hg until colorless perfusion fluid was obtained from the right atrium. Their brains were quickly isolated and sliced rostro caudally into serial 2 mm thick slices. Then, the brain slices were divided into the left and right hemispheres, and measurements of vascular permeability were made by comparing their weight with pre-weighed loci in the six slices. Samples from the slices were weighed and homogenized in tri-chloroacetic acid, following the formula of 1 g of brain tissue in 3 mL of 50% trichloroacetic acid for 24 h. They were then centrifuged at 10,000× *g* for 20 min, and the supernatant was diluted 1:3 with 96% ethanol [63]. A fluorescence detector was utilized at a 620 nm excitation wavelength (bandwidth 10 nm) and 680 nm emission wavelength (bandwidth 10 nm) for EB and at a 440 nm excitation wavelength (bandwidth 10 nm) and 525 nm emission wavelength (bandwidth 10 nm) for FNa [8,42]. A standard curve of FNa was established, with concentrations ranging from 0 to 3.5 × 10^−6^/g brain tissue (equivalent to the content in brain tissue) for the naive and control rats, and with concentrations ranging from 0 to 28 × 10^−6^/g brain tissue for the TBI groups. A standard curve of EB was established with concentrations ranging from 0 to 112 × 10^−6^/g brain tissue (equivalent to the content in brain tissue) for naive and control rats, and concentrations ranging from 0 to 2240 × 10^−6^/g brain tissue for the TBI groups. Measurements were performed on a plate with each well containing 100 μL of solution. All determinations were performed at least in duplicates.

### 4.7. Statistical Analysis

The statistical analysis was performed using the SPSS-24 software (SPSS Inc., Chicago, IL, USA). The Kolmogorov–Smirnov test was utilized to determine the appropriate tests for comparing the various parameters. A two-way ANOVA was employed to analyze the effects of brain trauma and sex. Levene’s test was conducted to verify the assumption of equal variances before performing t-tests. Group comparisons were evaluated using the Mann–Whitney test for non-parametric data and the two-sided t-test for parametric data. Correlations between MRI techniques for assessing BBB permeability and histological methods were determined using Spearman’s test for non-parametric data and Pearson’s test for parametric data. Normally distributed continuous variables were reported as mean ± SD, while non-parametric data were presented as median ± interquartile range. Statistical significance was defined as *p* < 0.05.

## 5. Conclusions

The main finding of this study is that the low- and high-molecular-weight complex markers demonstrated different dynamics of BBB disruption over a week in the same set of brain tissue using a TBI model. Additionally, we implemented our protocol for assessing BBB permeability using high- and low-molecular-weight complex markers in a single brain set, and we further determined the lower limit of sensitivity and demonstrated the accuracy of this method. We believe that this study will contribute to a deeper understanding of the mechanism of BBB disruption in the TBI model by providing a complete picture of BBB permeability at a critical time point for both low- and high-molecular-weight complexes, with the ultimate goal of safe and effective treatment of brain injury. The protocol we developed can be successfully used in studying BBB permeability in both acute and chronic neurodegenerative diseases, as well as in testing new treatment modalities for treating these diseases.

## Data Availability

Data are available upon reasonable request.
